# A Proving of Lachesis^200^

**Published:** 1890-09

**Authors:** S. Mills Fowler

**Affiliations:** Gainesville, Texas


### A PROVING OF LACHESIS$^{200}$.

S. Mills Fowler, M. D., Gainesville, Texas.

While a resident of St. Augustine, Florida, in 1888, I was called, November 24th, at about half-past ten A. M., to see Mrs. B. She was suffering from a left-sided sore throat, for which I gave her Lachesis$^{200}$, one dose, dissolved in about two spoonfuls of water, followed with placebo every two hours.

I was called again about half-past eight the next morning. The sore throat had all disappeared, but she had been suffering for hours "*with terrible pains in her shin bones.*"

November 26th, during my call, Mrs. B. said : "Doctor, didn't that medicine you gave me Saturday have something to do with those terrible pains in my legs? I never had anything like it before in my life."

Hering gives as a characteristic of Lachesis, "Much pain of an aching kind in the shin bones only."

I have, three different times since the above observation, verified this symptom clinically, and have come to regard it as "*the red string*" for Lachesis.

